# Natalizumab treatment reduces L-selectin (CD62L) in CD4^+^ T cells

**DOI:** 10.1186/s12974-015-0365-x

**Published:** 2015-08-12

**Authors:** Michela Spadaro, Marzia Caldano, Fabiana Marnetto, Alessandra Lugaresi, Antonio Bertolotto

**Affiliations:** Clinical Neurobiology Unit Neuroscience Institute Cavalieri Ottolenghi (NICO), Regione Gonzole 10, 10043 Orbassano, Turin Italy; AOU S. Luigi Gonzaga, Neurologia 2 - CRESM (Centro Riferimento Regionale Sclerosi Multipla), Regione Gonzole 10, 10043 Orbassano, Turin Italy; Department Neuroscience, Imaging and Clinical Sciences, University ‘G. d’Annunzio’, Chieti, Italy

**Keywords:** CD62L, Natalizumab, PML, Multiple sclerosis, IRIS

## Abstract

**Background:**

The purpose of this research was to validate the low expression of L-selectin (CD62L) in natalizumab (NTZ)-treated patients. CD62L is involved in rolling and transmigration of leukocyte cells. A correlation between CD62LCD4^+^ T cells low expression and progressive multifocal leukoencephalopathy (PML) development has been suggested in multiple sclerosis (MS) patients treated with NTZ.

**Methods:**

We performed a flow cytometric analysis on peripheral blood mononuclear cells (PBMC); we collected from 23 healthy donors and 225 MS patients: untreated (*n* = 19) or treated with NTZ (*n* = 113), interferon-beta (*n* = 26), glatiramer acetate (*n* = 26), fingolimod (*n* = 23) and rituximab (*n* = 18). We have also analysed two PML/IRIS (immune reconstitution inflammatory syndrome) patients and four longitudinal samples of a NTZ-treated patients before and during the development of a clinical asymptomatic magnetic resonance imaging (MRI) lesion confirmed as PML by cerebrospinal fluid (CSF) examination. Thirty-five NTZ-treated patients were studied longitudinally with three samples taken 4 months apart.

**Results:**

The NTZ-treated patients showed a lower percentage of CD62L (33.68 %, *n* = 113) than first-line treated patients (44.24 %, *n* = 52, *p* = 0.0004). NTZ effect was already clear during the first year of treatment (34.68 %; *p* = 0.0184); it persisted in the following years and disappeared after drug withdrawal (44.08 %). Three percent of longitudinally analysed patients showed a percentage of CD62LCD4^+^ T cells under a hypothetical threshold and one patient with asymptomatic PML belongs to a group which expressed low percentage of CD62LCD4^+^ T cells.

**Conclusions:**

Our research confirms that NTZ has a specific effect on CD62LCD4^+^ T cells consisting in decreasing of the number of positive cells. The low level of CD62L found in a clinically asymptomatic PML patient strengthens its potential usefulness as a biomarker of high PML risk in NTZ-treated patients. A larger study is required to better confirm the data.

**Electronic supplementary material:**

The online version of this article (doi:10.1186/s12974-015-0365-x) contains supplementary material, which is available to authorized users.

## Background

Natalizumab (NTZ) (Tysabri©, Biogen Idec Inc., Cambridge, MA) is a humanized monoclonal antibody which blocks the α4 subunit of the α4 integrins (α4β1 and α4β7) which is expressed on the surface of lymphocytes and leukocytes [1, 2]. α4β1 is required for endothelial adhesion, and it facilitates migration of peripheral blood lymphocytes to the brain via the blood-brain barrier [1]. NTZ showed its efficacy in the multiple sclerosis (MS) treatment by reducing the annual relapse rate in two clinical trials by 68 % [3, 4]. This monoclonal antibody is currently considered to be one of the best therapeutical options for patients with relapsing-remitting MS (RRMS) not responding to traditional therapies [5].

The most fearsome clinical complication of NTZ treatment is progressive multifocal leukoencephalopathy (PML), a John Cunningham virus (JCV) infection of the brain [6]. PML is a demyelinating condition characterized by the degenerative loss of cerebral white matter after infection by JCV, a normally latent polyomavirus which becomes virulent in the presence of immunosuppression [7]. During therapy with NTZ, PML risk can be stratified depending on three risk factors: anti-JCV antibody status and level, treatment length (more or less than 2 years) and prior immunosuppressive therapy [8, 9]. It is important to identify other factors allowing better identification of MS patients at high risk for PML. Schwab and collaborators [10] investigated the expression of T lymphocytes surface markers in NTZ-treated patients before PML development. Their investigation suggested L-selectin (CD62L) expressed on CD4^+^ T cells might help identify MS patients at high risk for PML, since all the eight patients who developed PML had a previous blood sample showing a level of CD62L expression below a hypothetical threshold [10].

The aim of this study was to investigate whether the expression of CD62L is lower in NTZ-treated patients than in patients treated with other drugs and to evaluate levels of CD62L in a subset of NTZ-treated patients throughout treatment.

## Methods

### Standard protocol approval, registration and patients consent

We obtained peripheral blood samples from healthy individuals and from subjects with RRMS after they signed a written informed consent approved by the Ethics Committee of San Luigi Gonzaga University Hospital (Ethic approval number 7777, March 25, 2013). The blood samples were collected in 6 ml ethylenediaminetetraacetic acid (EDTA) Vacutainers (BD Biosciences, Milan, Italy) and processed within the following 4 h. This study was performed according to good clinical practice and the Declaration of Helsinki.

### Patients and healthy controls

We enrolled 225 patients with RRMS, aged 39 ± 10 years. We excluded subjects with concomitant endocrine and metabolic disorders. Nineteen patients were untreated (UT), 113 were treated with NTZ (300 mg iv every 28 days, duration 1–86 months), 26 were treated with interferon-beta (IFNb), 26 were treated with glatiramer acetate (GA), 23 were treated with fingolimod (FTY) and 18 were treated with rituximab (RTX). At the time of enrolment, clinically definite MS was diagnosed using the revised McDonald’s criteria [11]. Age- and sex-matched healthy donors (HD, *n* = 23) with no previous history of any neurologic or immune-mediated disease served as controls (Table 1). We have also analysed samples from two PML/IRIS (immune reconstitution inflammatory syndrome) patients, who had discontinued NTZ treatment, and we collected other four samples during 9 months from a patient who developed clinical asymptomatic PML while under NTZ. All patients underwent periodic clinical and magnetic resonance imaging (MRI) evaluations.

**Table 1:** Patient characteristics

Group	Women/men (% women)	Age: years ± SD (range)	Mean EDSS at the sampling (interval)	Treatment duration (months or infusions ± SD)	Previous treatment
HD (*n* = 23)	18/5 (78 %)	33 ± 11 (20–60)	n.d.	n.d.	n.d.
UT (*n* = 19)	12/7 (63 %)	41 ± 8 (27–57)	1.3 (0–3)	n.d.	n.d.
GA (*n* = 26)	16/10 (62 %)	44 ± 11 (22–60)	1.9 (0–8.5)	45 ± 38 (1–122)	13/26 IFNb; 9/26 nv; 4/26 IS
IFNb (*n* = 26)	15/11 (58 %)	36 ± 11 (24–65)	1.6 (0–5)	51 ± 48 (3–147)	25/26 nv; 1/26 GA
NTZ (*n* = 113)	76/37 (67 %)	37 ± 9 (19–60)	2.9 (0–8)	35 ± 24 (1–86)	52/113 IFNb; 23/113 GA; 13/113 IS; 12/113 nv; 6/113 FTY; 7/113 other treatments
RTX (*n* = 18)	15/3 (83 %)	41 ± 10 (30–65)	4.4 (2–7)	1.25 (1–3)	1/18 IFNb; 1/18 GA; 5/18 FTY; 9/18 NTZ; 2/18 n.d.
FTY (*n* = 23)	15/8 (65 %)	41 ± 9 (21–57)	3.1 (0–6.5)	15 ± 8 (1–29)	1/23 nv; 8/23 NTZ; 5/23 GA; 5/23 IFNb; 2/23 IS; 2/23 other treatments

*HD* healthy donors, *UT* untreated multiple sclerosis patients, *GA* glatiramer acetate, *IFNb interferon-beta*, *NTZ* natalizumab, *RTX* rituximab, *FTY* fingolimod, *n.d.* not determined or not applicable, *EDSS* Expanded Disability Status Scale, *nv* naïve patient, *IS* immunosuppressive drug

### Blood samples and bio-banking

CRESM (Centro di Riferimento Regionale per la Sclerosi Multipla) at University Hospital San Luigi, Orbassano, Italy, houses a biological bank collecting samples from HD and MS patients. The bio-bank stores biological samples (cerebrospinal fluid (CSF), RNA, DNA, sera, plasma and peripheral blood mononuclear cells (PBMC)) for research purposes. For this research, blood samples were drawn just before NTZ-injection or during planned routine visits in patients treated with other drugs.

PBMC were isolated from EDTA-treated blood by Lymphoprep density gradient centrifugation. Then, cells were cryopreserved in liquid nitrogen using freezing medium: 60 % RPMI 1640 medium (Invitrogen Life Technologies, Grand Island, NY, USA), 30 % heat-inactivated foetal bovine serum (FBS, Invitrogen Life Technologies) and 10 % dimethyl sulfoxide (DMSO, Sigma-Aldrich, St Louis, MO). All assays were performed on fresh frozen cells. After gentle thawing at 37 °C, the cells were immediately added to 5 mL RPMI 1640 supplemented with 10 % heat-inactivated FBS and centrifuged to remove DMSO. The samples were re-suspended in RPMI 1640 medium supplemented with 10 % heat-inactivated FBS and counted.

### Flow cytometry

Non-specific sites of 2.5 × 10^5^ cells were blocked with rabbit immunoglobulins G (IgG, Sigma-Aldrich). Then, the cells were incubated with fluorochrome-conjugated monoclonal Ab (mAb) and isotype-matched negative controls for 20 min at 4 °C. The following mAbs were used: anti-human CD3 APC-Vio770 (Miltenyi Biotec, Bergisch Gladbach, Germany), anti-human CD4 PE-Cy7 and anti-human CD62L FITC allophycocyanin (both from BD Pharmingen, San Diego, CA) and isotype-matched mAb (Miltenyi Biotec). After staining, the cells were washed and re-suspended in PBS (Sigma-Aldrich) supplemented with 0.2 % bovine serum albumin and 0.01 % sodium azide (both from Sigma-Aldrich). The samples were collected and analysed using a CyAn ADP, running Summit 4.3 analysis software (Beckman Coulter, Brea, CA, USA). The living cells identified by propidium iodide (Sigma-Aldrich) exclusion were gated according to their light scatter properties to exclude cell debris. Quality experiments have been performed (Additional file 1: Figure S1) in order to evaluate whether the freeze-thawing cycle could affect the quantification of CD62L protein expression and to check the reproducibility of the flow cytometric procedure and the stability of the expression of CD62L on CD4^+^ T cells in frozen samples.

### Statistical analysis

Statistical analysis was performed using GraphPad Prism software (GraphPad Software, version 4; San Diego, CA, USA). The differences between the two groups were calculated with an unpaired, two-tailed, nonparametric Mann-Whitney *U* test. The Student’s paired *t* test was used to evaluate differences in the longitudinal studies.

## Results

### Expression of CD62L on CD4^+^ T cells in healthy donors and in MS patients untreated or treated with IFNb, GA, NTZ, FTY and RTX

**Fig. 1:**
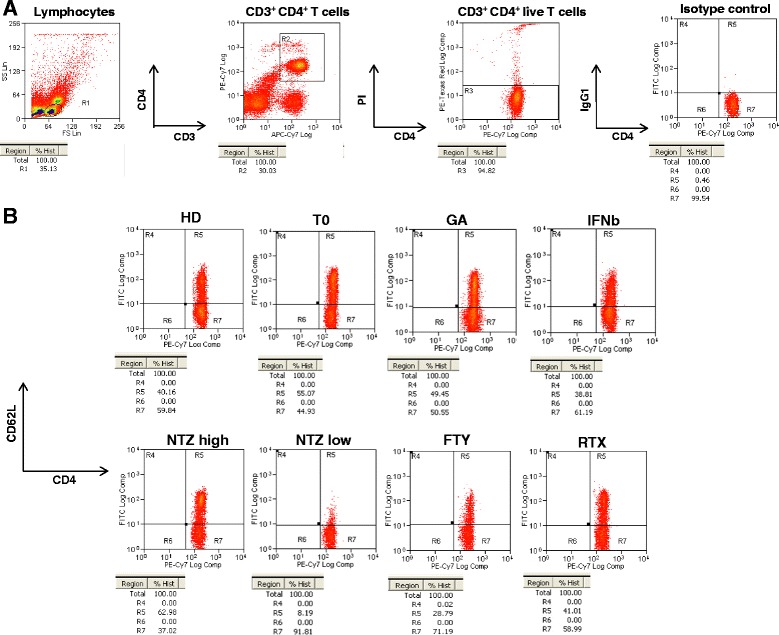
Flow cytometry gating strategy. **a** A R1 gate was set on lymphocytes in the forward scatter (FS) versus side scatter (SS) *dot plot*. Lymphocyte cells were then gated (R2) for the double positivity CD3^+^CD4^+^ markers, and finally, living cells were identified by propidium iodide negativity (R3). A quadrant region for CD62LCD4^+^ T cells was then created using an isotype control. **b** Different *dot plots* from control subjects or MS treated patients. A single subject for each treatment was shown

**Fig. 2:**
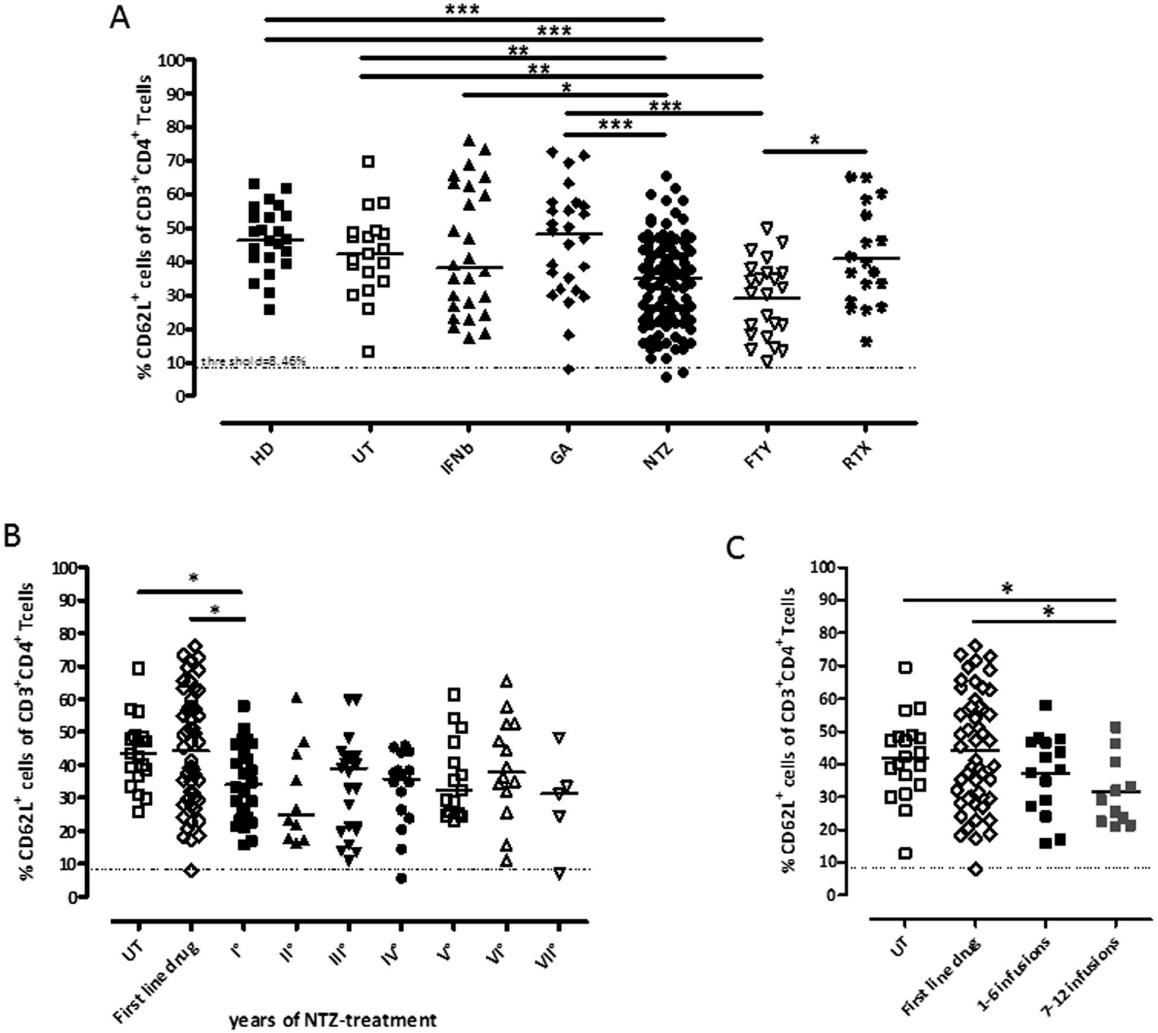
CD62L evaluation in HD and MS patients. **a** Comparison of different treatments. CD62L evaluation in HD (23, *solid square*), UT (19, *open square*), IFNβ (26, *solid triangle*), GA (26, *solid diamond*), NTZ (113, *solid circle*), FTY (23, *open triangle*) and RTX (18, *asterisk*) groups. The *dotted line* displays the tentative threshold (8.46 %), the *solid line* indicates the mean. **b** Stratification of NTZ analysis by year of treatment. **c** The first and second semesters of the first year of treatment. CD62L in the first semester, 1–6 months (15 patients; *black square*), the second semester, 7–12 months (11 patients; *grey square*), and in first-line drug-treated patients (52 patients, *open diamond*). The statistical differences were calculated with the Mann-Whitney *U* test

To verify the impact of NTZ on the expression of CD62L on the CD4^+^ T cell population, we tested our cohort at least 15 days after freezing, as described by Schwab and collaborators [10]. The strategy of CD62LCD4^+^ T cells gating is described in Fig. 1. Compared to HD (46.75 % ± 9.8; mean ± SD) the expression of CD62L on CD4^+^ T cells was significantly lower in patients treated with NTZ (33.68 % ± 12.7; *p* < 0.0001) (Fig. 2a). We have also analysed the expression of this molecule on CD4^+^ T cells from UT naïve MS patients or from patients treated with GA, IFNb, FTY and RTX. In patients treated with GA, IFNb and UT, the expression of CD62L was higher than in the NTZ-treated patients: 47.03 % ± 14.7 (*p* = 0.0001) in the GA-treated patients, 42.95 % ± 18.9 (*p* = 0.0454) in the IFNb-treated patients and 43.5 % ± 10.8 (*p* = 0.0028) in the UT patients (Fig. 2a). The expression of CD62L was extended to two second-line disease-modifying treatments (DMTs), FTY and RTX, which had not been studied by Schwab and collaborators. The expression of CD62L in HD, UT, IFNb and GA was not significantly different from the RTX group (41.11 % ± 14.63), whereas it was higher than in the FTY group (28.76 % ± 11.42; HD: *p* < 0.0001, UT: *p* = 0.0022, GA: *p* = 0.0002, respectively) (Fig. 2a). The comparison of CD62L expression among second-line treatment patients showed the percentage of CD62L was higher in the RTX group than in the FTY (28.76 % ± 11.42; *p* = 0.0121), but it was not statistically different from the NTZ group (Fig. 2a).

Considering a low percentage of CD62LCD4^+^ might represent a biomarker of the risk to develop PML, a tentative threshold of risk was set up at 8.46 % representing the mean (34.08 %) of the long-term NTZ-treated patients (>18 infusion) minus two times the SD (12.81). Applying this threshold, 2 out of 80 long-term treated patients (2.5 %) in the NTZ-treated group showed an expression of CD62L below the threshold; both patients had been treated with NTZ for more than 2 years (Fig. 2b). One patient treated with GA showed a percentage of CD62L below the threshold.

To evaluate if the duration of NTZ treatment influences the level of CD62L, NTZ-treated patients were stratified according to the number of infusions. NTZ significantly reduced the expression of CD62L versus the UT patients (*p* = 0.0200) and first-line therapies (*p* = 0.0184) (Fig. 2b) already during the first year of treatment; the reduction of the expression began during the first semester of the treatment, and it reached a statistically significant difference in the second semester (UT *p* = 0.0226; first-line treatment *p* = 0.0220) (Fig. 2c). The decreased CD62L expression persisted in the following years with no statistically significant fluctuations (Fig. 2b).

### Longitudinal expression of CD62L during NTZ treatment

**Fig. 3:**
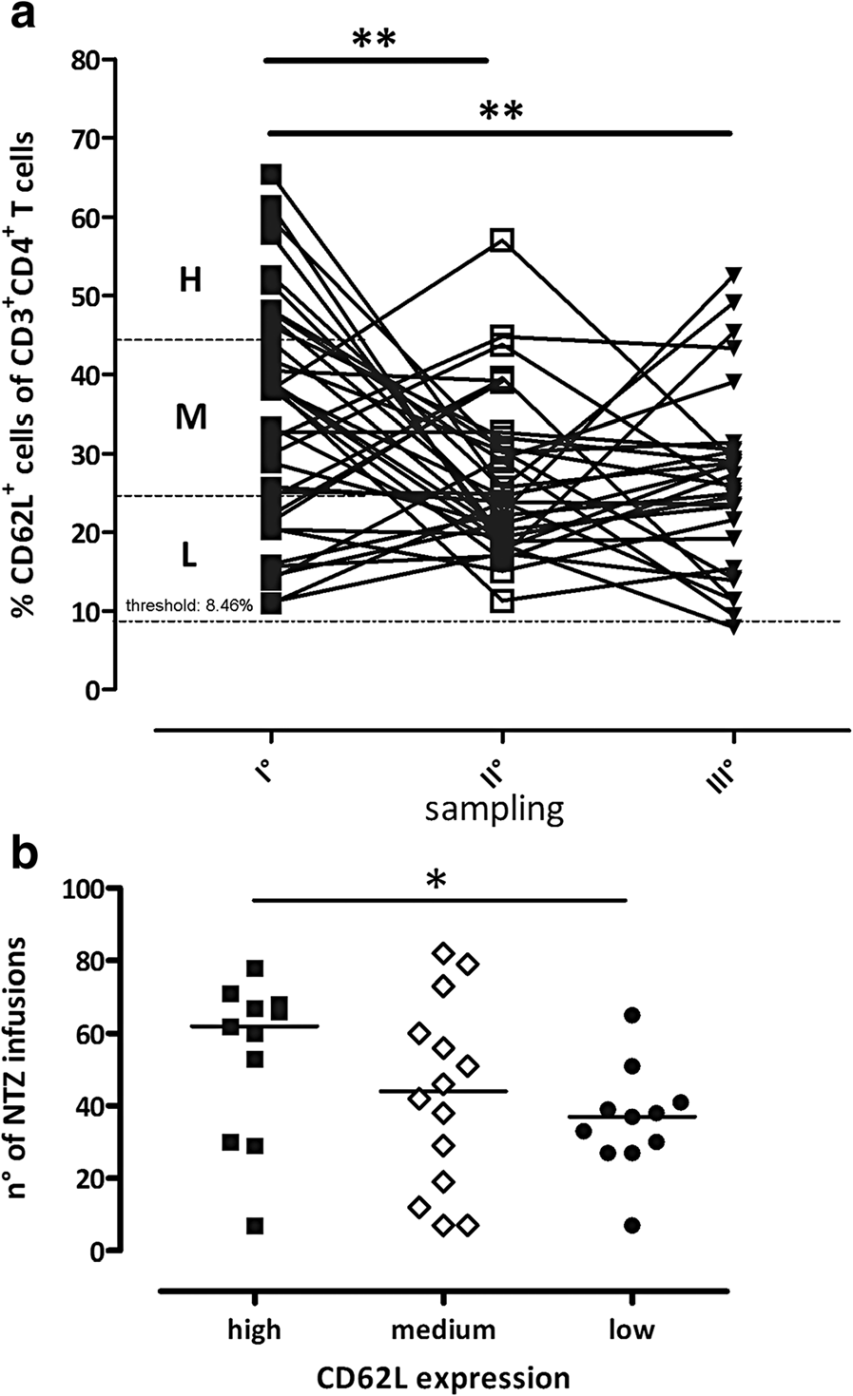
Longitudinal study of the NTZ-treated patients. **a** Thirty-five NTZ patients were analysed three times during treatment: first, second and third sampling. The overall results of the first sample were divided into three regions. High, low and medium indicate respectively the region above the 75th percentile (*H*; *black square*), that below the 25th percentile (*L*; *solid circle*) and the region between *H* and *L* (*M*; *open diamond*). The statistical differences were calculated with Student’s paired *t* test. **b** Number of infusions in the three different groups. The statistical differences were calculated using Mann-Whitney *U* test

CD62L was measured in 35 patients during NTZ treatment (7–82 infusions), three times at four monthly intervals to evaluate the longitudinal fluctuations of L-selectin expression. The patients were selected in order to explore the full range of CD62L expression, from low to high. In particular, the first sample was categorized as low (L) if below the 25th percentile (CD62L <24 %; 10 patients), as medium (M) if between the 25th and the 75th percentile, (CD62L >24 % and <43 %; 14 patients) and as high (H) if above the 75th percentile (CD62L >43 %, 11 patients) (Fig. 3a). The categorization in the H, M and L groups was not strictly dependent on the number of infusions as the H group presented a number of infusions (median 62, range 7–78) similar to the M group (median 44, range 7–82), although higher than the L group (median 33, range 7–65, *p* = 0.0302) (Fig. 3b). When we considered the second and third draw, the percentage of CD62L was significantly lower than in the first one (*p* = 0.0087, *p* = 0.0094, respectively) with no differences between the second and the third tap (Fig. 3a). The longitudinal study showed that 1 out of 35 patients (3 %) presented one of the three samples below the tentative threshold.

### Expression of CD4CD62L^+^ after NTZ discontinuation

Six patients discontinued NTZ treatment because they considered the risk of PML too high (Table 2). CD62L was studied during NTZ treatment, from 16 to 71 infusions, and 3 to 5 months after withdrawal (Table 2). Three out of six patients showed a low level of CD62L^+^ during treatment, which reverted to UT values after discontinuation as shown in Table 2. The expression of CD62L remained almost unchanged in two patients who had values similar to the mean of the NTZ group, while in patient #6, CD62L expression decreased in 32 % 3 months after discontinuation, although it was not below the tentative threshold and remained in the medium group between 25th and 75th percentile. The patients were monitored by MRI to evaluate the status disease after NTZ discontinuation. Only one patient (pt #3) with the unvaried CD62L expression had a relapse (Table 2).

**Table 2:** Clinical features and percentage of CD62L positive CD62LCD4^+^ T cells in 6 patients who discontinued the NTZ treatment

		During NTZ infusion		After NTZ withdrawal			
	JCV index	Number of infusion	CD62LCD4 (%)	Number of months	CD62LCD4 (%)	MRI	Relapse
Pt #1	3.498	40	5.65	5	49.07	Neg	None
Pt #2	2.488	16	16.34	3	56.48	Neg	None
Pt #3	2.696	20	35.44	4	32.21	Pos	1
Pt #4	2.034	71	44.50	3	42.32	Neg	None
Pt #5	1.366	66	15.83	4	56.59	Neg	None
Pt #6	2.304	32	41.18	3	27.78	Neg	None

*Neg* unvaried number of lesions compared to previous MRI

### Expression of CD62L in a single patient during the development of asymptomatic PML

**Fig. 4:**
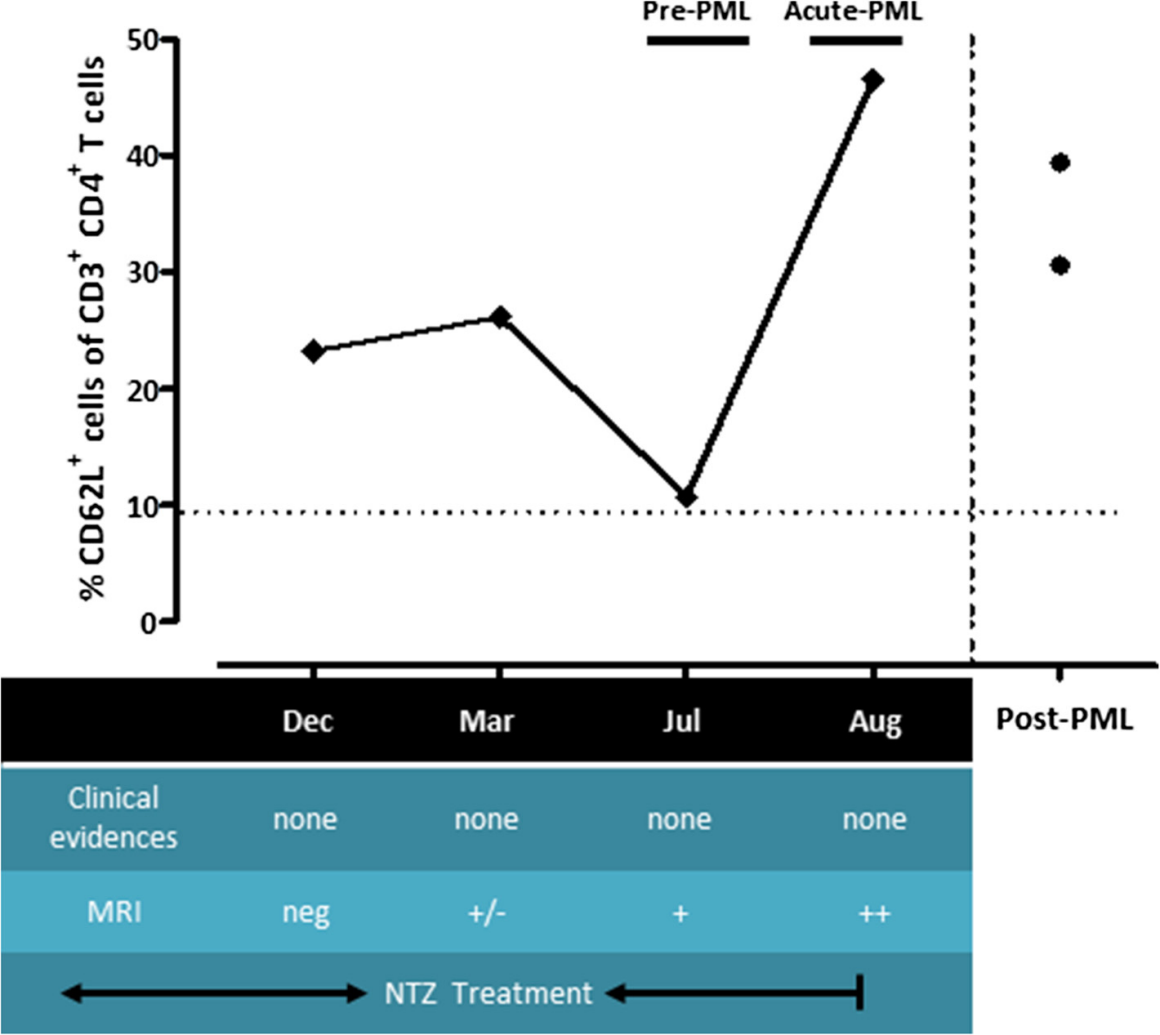
Longitudinal analysis of CD62L expression in one patient who developed PML and CD62L expression in two other patients during the MS reactivation/IRIS phase of PML. The PML patient was analysed by MRI and by flow cytometry four times during treatment. The value (*solid diamond*) nearby the threshold (*dotted line*) was found in July, when MRI was suggestive of PML. In August, PML was diagnosed by detection of 80 copies of JCV in the cerebrospinal fluid, in the absence of clinical manifestation. The *black circles* identify two patients in the reactivation/IRIS phase, 4 months after PML diagnosis and NTZ withdrawal

We had the opportunity to examine one NTZ-treated female patient four times in December 2013, March, July and August 2014 in the meanwhile she was developing an asymptomatic PML. At the same time as CD62L expression analysis, the patient underwent neurological examination and MRI scans, which were reported as negative for PML in December and March and strongly suggestive for PML in July. In August, the patient was admitted to the hospital: the neurological examination was negative, a new MRI showed an enlargement of the PML-suspected lesion and 80 JCV DNA copies per ml were detected in the cerebrospinal fluid (Fig. 4). A diagnosis of asymptomatic PML was made, and NTZ was discontinued. A re-evaluation of the MRI scan performed in March revealed a small T2 lesion in the occipital right lobe, where the larger lesion was subsequently detected in July (Fig. 4). CD62L showed a value of 23.21 % in December and of 26.14 % in March; the blood tap in July showed a value of 10.65 % which is nearby the tentative threshold of 8.46 % and belongs to the L group. When the diagnosis of asymptomatic PML was made in August, the value of CD62L was 46.52 % (Fig. 4).

### Expression of CD62L in PML/IRIS cases

We also had the possibility to evaluate the percentage of CD62L in two cases of PML, during the IRIS phase, 4 months after NTZ treatment had been discontinued: CD62L values were similar to the mean value of the NTZ-treated group (Fig. 4).

## Discussion

The main goal of this research was to test whether NTZ treatment has a specific impact on CD62L expression in CD4^+^ T cells as suggested by Schwab and collaborators [10]. This issue is relevant as low levels of CD62L were suggested as a potential marker for PML risk stratification in NTZ-treated patients [10].

The CD62L is a key adhesion molecule which regulates both the migration of leukocytes at sites of inflammation and the recirculation of lymphocytes between blood and lymphoid tissues. In our work, we analysed separately the first-line drugs (IFNb and GA). The data are consistent with those already reported since the patients treated with NTZ showed a lower percentage of CD62LCD4^+^ T cells than the patients treated with IFNb (*p* = 0.0454) and GA (*p* = 0.0001). Although data in literature show that the GA had no impact on the expression of the T cell surface activation markers such as CD62L in animal model [12], in our study, a GA-treated patient showed a low percentage of CD62LCD4^+^ T cells for no apparent reason. Monitoring the medical record, 4 months later CD62L analysis, the patient had a serious infection resulting in prolonged hospitalization. This data shows how this might be a correlation between the low percentage of CD62L and the subsequent development of infections even though this is not closely related to MS. We have also studied in our research the impact of two second-line DMTs on CD62L. RTX does not influence CD62L expression on CD4^+^ T cells, since we have not detected any differences with HD controls, UT, GA- and IFNb-treated patients (Fig. 2). FTY induced a decreased CD62L expression, similarly to NTZ (Fig. 2). The peculiarity of CD62L is that it acts as a “homing receptor” for leukocytes to enter secondary lymphoid tissues via high endothelial venules. The function of FTY is the segregation of lymphocytes in the lymph nodes. Previous reports showed FTY mainly influences naïve and central memory cells [13], and probably for these reasons, a high percentage of CD62LCD4^+^ T cells are absent in the blood of our patients. Furthermore, different patients were previously treated with NTZ and this could influence the expression of CD62L on CD4^+^ T cells. The phenomenon we observed is very interesting and deserves a dedicated study to evaluate the expression of CD4CD62L^+^ T cells on a broader population of FTY-treated patients and its stability during time.

In Fig. 2, we took a picture of the first sampling by evaluating all the available patients we had at that time. Then, we stratified the patients according to the number of given infusions, and we noticed only the first year was statistically different, while the next was no longer (Fig. 2b). These data suggest an implication of NTZ in the variation of CD62L expression on CD4^+^ T cells. To evaluate the effect of NTZ on the expression of CD62L in the first year, we have separated the first 6 months of treatment and the second ones. Our results show a persistent CD62L low expression if you continue the treatment, and it disappears after treatment discontinuation. In the group of patients who had discontinued NTZ, the patients who showed a significant increase of CD62L percentage belonged to the 25th percentile group (pt #1, pt #2, pt #5). Moreover, the patient (pt #6) who had a decrease of CD62L expression remained in the medium group. Only one patient (pt #3), who had an unchanged value of CD62L, had a relapse 4 months after NTZ suspension (MRI pos, see Table 2): this is an indication that CD62L is a marker of infections and not of the MS relapse activity. However, all patients were monitored by MRI 2–3 months after discontinuation, and this exam was unchanged compared to the previous result in five patients out of six.

In the whole subsets of patients, freeze-thawed cells showed a lower percentage of CD62L expression than fresh cells, but the decrease was more relevant in NTZ-treated patients (Additional file 1: Figure S1). Our data confirmed prolonged interaction between NTZ and PBMC expressing α4 integrins induces increased cell activation causing easier shedding of CD62L [14, 15] or reduced cell viability.

As freeze-thawing strongly decreases the percentage of PBMC CD62L^+^ cells, a phenomenon already observed on polymorphonuclear leukocytes [16], the procedures of PBMC collection, banking and freeze-thawing are crucial and small variations could explain the lower percentage of CD62LCD4^+^ T cells we found in comparison with the Schwab study [10]. Staining and gating of positive cells do not seem to influence the results as the reproducibility of flow cytometry quantification of CD62L-positive cells was very good in the same frozen sample (Additional file 1: Figure S1).

The seminal work on the role of CD62L as a biomarker for PML risk stratification showed all the eight patients, who later developed PML, had a value of CD62L below a tentative threshold [10]. In this work, the longitudinal data of a patient who later developed PML support the role of CD62L as a risk biological marker. In order to get a precise comparison with the work of Schwab and colleagues [10], we should calculate the threshold value as the mean minus twice the standard deviation of the percentage of CD62L in long-term NTZ-treated patients. A value of CD62L nearby to the tentative threshold was detected in the clinically asymptomatic phase of the disease, and a value in the lower 25th percentile was detected when MRI was judged negative for PML (Fig. 4). This observation opens a question about the establishment of threshold risk. In this research, we found a correlation between the low percentage of CD62L and the development of a severe infection. A greater number of observations are required to establish a threshold, and it is also necessary to evaluate critically whether the values obtained could be arranged in the 25th percentile or below the long-term NTZ-treated patients threshold or if it is better to calculate the threshold based on the first-line drug group since the majority of NTZ patients (75 patients out of 113, Table 1) starts NTZ after the first-line drug treatment.

Previous studies have shown that NTZ-treated MS patients have an increased pool of T cells and other lymphocytes in the blood compartment [17, 18] which may be enriched in T cells with an increased activation state. The percentage of CD62L in each patient fluctuates in time; in fact, the longitudinal measurements in the same patients showed 3 % of the patients tested three times 4 months apart had one single value below the empirically established threshold (Fig. 3), and the value of CD62L decreased during time. Considering PML risk increases with time on treatment [19], in our analysis, we found patients with a percentage of CD62L below threshold in the fourth and seventh year (Fig. 2b). The patients with CD62L low expression will be closely monitored by MRI and CD62L analysis. In addition to the existing parameters, the analysis of CD62L could be another parameter to evaluate patients’ health and their risk to develop PML.

## Conclusions

Our investigation confirms that a low percentage of CD62LCD4^+^ T cells could represent a potential biomarker for PML risk stratification. A multicentre study enrolling a larger number of patients using homogeneous flow cytometry protocol is mandatory to establish a PML risk threshold and to define the timing of CD62LCD4^+^ quantification.

## Additional file

Additional file 1: Figure S1
**Set up procedure**. A Expression of CD4^+^ T cells. PBMCs were analysed before (solid symbol) and after (open symbol) freezing in group A (six patients, four HD and two first-line treated MS; squares) and NTZ (five patients; diamonds). B Expression of CD62L on CD4^+^ T cells. PBMCs were analysed before (solid symbol) and after (open symbol) freezing in group A (six patients, four HD and two first-line treated MS; squares) and NTZ (five patients, diamonds). After freezing CD62L expression decreased by 31 % in the CTRL group and by 33 % in the NTZ group. C Analysis of stability and reproducibility. The frozen samples from the same patient were evaluated for CD62L expression in two different work sessions 3 months apart. The statistical differences were calculated with Student's paired *t* test.

## Abbreviations

CSF: cerebrospinal fluid
CRESM: Centro di Riferimento Regionale per la Sclerosi Multipla
DMTs: disease-modifying treatments
DMSO: dimethyl sulfoxide
EDTA: ethylenediaminetetraacetic acid
FBS: foetal bovine serum
FTY: fingolimod
GA: glatiramer acetate
HD: healthy donors
IFNb: interferon-beta
IRIS: immune reconstitution inflammatory syndrome
JCV: John Cunningham virus
MRI: magnetic resonance imaging
MS: multiple sclerosis
NTZ: natalizumab
PBMC: peripheral blood mononuclear cells
PML: progressive multifocal leukoencephalopathy
RRMS: relapsing-remitting multiple sclerosis
RTX: rituximab
UT: untreated
