# Supplementary figures and images for: Natalizumab treatment reduces L-selectin (CD62L) in CD4+ T cells
Source: J Neuroinflammation. 2015 Aug 12;12:146. doi: 10.1186/s12974-015-0365-x (PMC4532246; doi:10.1186/s12974-015-0365-x)

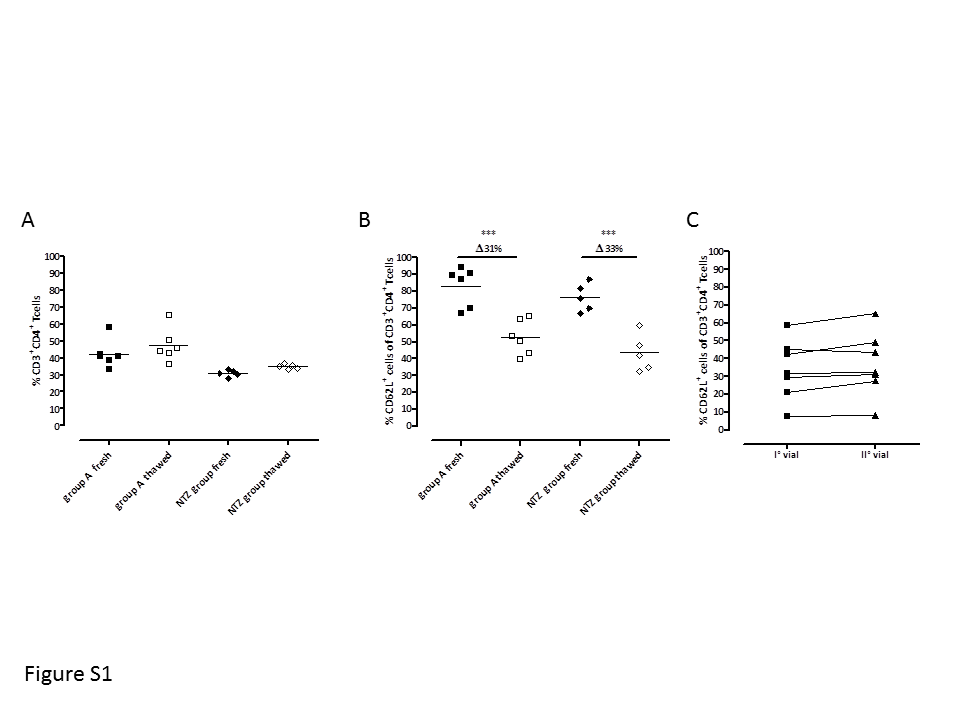

Supplement: Additional file 1: Figure S1 — Set up procedure. A Expression of CD4+ T cells. PBMCs were analysed before (solid symbol) and after (open symbol) freezing in group A (six patients, four HD and two first-line treated MS; squares) and NTZ (five patients; diamonds). B Expression of CD62L on CD4+ T cells. PBMCs were analysed before (solid symbol) and after (open symbol) freezing in group A (six patients, four HD and two first-line treated MS; squares) and NTZ (five patients, diamonds). After freezing CD62L expression decreased by 31 % in the CTRL group and by 33 % in the NTZ group. C Analysis of stability and reproducibility. The frozen samples from the same patient were evaluated for CD62L expression in two different work sessions 3 months apart. The statistical differences were calculated with Student's paired t test. [file 12974_2015_365_MOESM1_ESM.tif]
